# Muse Cells: Nontumorigenic Pluripotent Stem Cells Present in Adult Tissues—A Paradigm Shift in Tissue Regeneration and Evolution

**DOI:** 10.1155/2016/1463258

**Published:** 2016-12-14

**Authors:** Ariel A. Simerman, Julia D. Phan, Daniel A. Dumesic, Gregorio D. Chazenbalk

**Affiliations:** Department of Obstetrics and Gynecology, David Geffen School of Medicine at The University of California, Los Angeles, Los Angeles, CA 90095, USA

## Abstract

Muse cells are a novel population of nontumorigenic pluripotent stem cells, highly resistant to cellular stress. These cells are present in every connective tissue and intrinsically express pluripotent stem markers such as Nanog, Oct3/4, Sox2, and TRA1-60. Muse cells are able to differentiate into cells from all three embryonic germ layers both spontaneously and under media-specific induction. Unlike ESCs and iPSCs, Muse cells exhibit low telomerase activity and asymmetric division and do not undergo tumorigenesis or teratoma formation when transplanted into a host organism. Muse cells have a high capacity for homing into damaged tissue and spontaneous differentiation into cells of compatible tissue, leading to tissue repair and functional restoration. The ability of Muse cells to restore tissue function may demonstrate the role of Muse cells in a highly conserved cellular mechanism related to cell survival and regeneration, in response to cellular stress and acute injury. From an evolutionary standpoint, genes pertaining to the regenerative capacity of an organism have been lost in higher mammals from more primitive species. Therefore, Muse cells may offer insight into the molecular and evolutionary bases of autonomous tissue regeneration and elucidate the molecular and cellular mechanisms that prevent mammals from regenerating limbs and organs, as planarians, newts, zebrafish, and salamanders do.

## 1. Introduction

Stem cell regulation of growth and regrowth in animals is rooted in an elusive mechanism, confounding the world's scientific leaders and giving rise to a wide variety of hypotheses and refutations over the course of the last century. The most intriguing piece of the puzzle to date is a mammalian shortcoming with regard to autonomous regeneration. What prevents mammals from regenerating limbs and organs, as other organisms do?

Studies on embryonic stem cells (ESCs), which have the ability to differentiate into all types of cells, have been geared towards not only answering this question, but also generating these processes in mammals. On the other hands, induced pluripotent stem cells (iPSCs), reprogrammable pluripotent stem cells generated through artificial manipulation, are unsuitable to study regeneration from an evolutionary standpoint. Various nonreprogrammed pluripotent stem cell populations have also been put forth to answer this call. Multipotent adult progenitor cells (MAPCs), isolated from bone marrow, have exhibited a regenerative capacity* in vivo* [1]. Human marrow-isolated adult multilineage inducible (MIAMI) cells [2], very small embryonic-like stem cells (VSELs) [3], and unrestricted somatic stem cells (USSCs) [4] exhibit pluripotency in their own right. Stimulus-triggered acquisition of pluripotency (STAP) cells, perhaps the most promising among their peers in their capacity for reprogramming, have been repudiated entirely. Thus, the scientific community is in dire need of a different, more primal model to explain these phenomena and elucidate future avenues of investigation.

**Figure 1:**
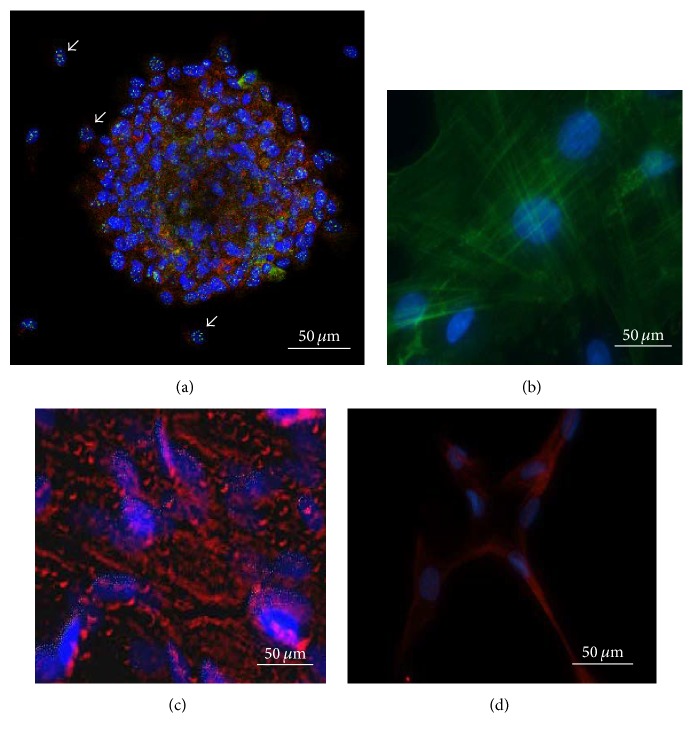
Muse-AT cells can differentiate into mesodermal, endodermal, and ectodermal cell lineages. Muse-AT cells can grow in suspension, forming spheres or cell clusters as well as individual cells (see white arrows) both expressing characteristic pluripotent stem cell markers, such as Oct4 (Texas Red) and TRA-1-60 (Green Fluorescence GFP), while nuclei were stained with DAPI (blue) (a); myogenic differentiation medium; the formation of myocytes (mesodermal origin) was detected using an anti-human MSA antibody (b); hepatogenic differentiation medium; formation of hepatocytes (endodermal origin) was detected using an anti-cytokeratin 7 antibody (c); neural differentiation medium; neural-like cells (ectodermal origin) were detected by immunofluorescence using an anti-human MAP2 antibody (d). Nuclei were stained with DAPI (blue) (original magnification 600x). (b)–(d) From Figures 3(C), 4(C), and 5(D) [5].

**Figure 2:**
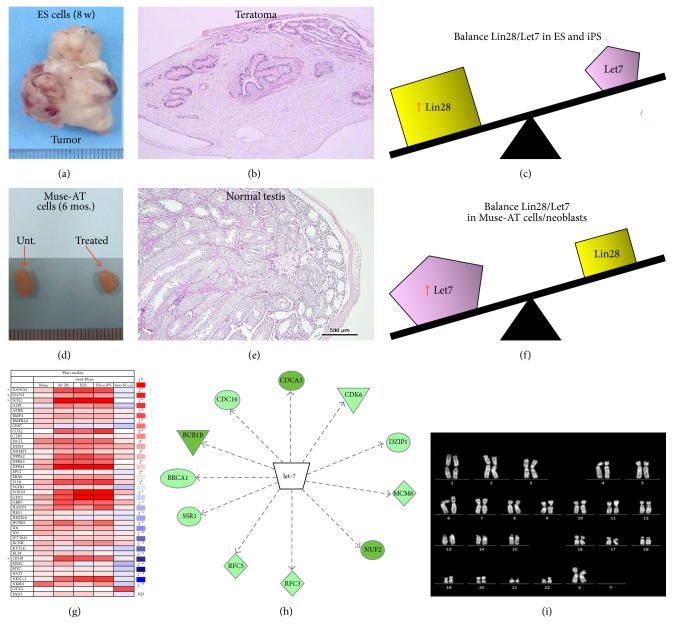
Properties of Muse-AT cells. Nontumorigenicity of Muse-AT cells. Embryonic stem (ES) cells injected into immunodeficient mice (SCID mice) testes, formed teratomas within 8 to 12 weeks (a). Histological analysis showed that the teratoma contained muscle tissue, intestine-like structure, and keratinized skin (b). Muse-AT cells transplanted into testes did not form teratomas even 6 months after injection, similar to untreated testes (d). Testis injected with Muse-AT cells maintained normal structure (e). Yin-Yang balance between Let7 and Lin28: ES and IPS expressed much higher levels of Lin28 versus Let7 (c). Muse-AT cells and neoblasts expressed much higher levels of Let7 versus Lin28 (f). Differences in expression of pluripotent stem cell genes between Muse cells, ES, iPS, and non-Muse cells determined by qRT-PCR (g). MicroRNA Let-7 is an upstream regulator of* CDA3*,* CDC16*,* DZIP1*,* SSR1*,* RFC3*,* RFC5*,* MCM6*,* NUF2*,* BRCA1*,* BUB1B*, and* CDK6* (h). Normal karyotype of human Muse-AT cells is indicated by 23 intact pair of chromosomes with a pair XX chromosome indicating female origin of the cells (i). (a)-(b) From Figure 3: [6]. (g) From Table 1 [7]. *∗* indicates pluripotent stem cell markers.

Recently, a novel population of pluripotent stem cells, highly resistant to severe cellular stress, named Multilineage Differentiating Stress Enduring Cell (Muse cells), has been discovered. Muse cells grow in suspension as cell clusters reminiscent of embryonic stem cells (Figure 1(a)). Muse cells intrinsically express pluripotency markers including SSEA3, TRA1-60, Nanog, Oct3/4, and Sox2, although at very low levels in comparison with ESCs and iPSCs (Oct3/4, <100-fold; Nanog and Sox2, <1000-fold) (Figure 2(g)) [5–8]. Muse cells differentiate into cells from the three embryonic germ layers both spontaneously and under media-specific induction (Figures 1(b)–1(d)) [5, 7–9]. Interestingly, Wakao et al. have shown that human dermal fibroblasts Muse cells (SSEA-3+, ~1% of the total population), but not non-Muse cells (SSEA-3−, ~99% of the population population), have the capacity to become iPSCs in the presence of four Yamanaka factors (Oct3/4, Sox2, Klf4, and c-Myc). This lends support to the elite model rather than the stochastic model of iPSCs generation [7].

Muse cells are “natural cells” present in all connective tissues of the body. The existence of Muse cells has been demonstrated in bone marrow, skin cells, and adipose tissue by seven independent groups worldwide [5, 8, 10–14]. They exist normally in a quiescent state and are activated when exposed to conditions of severe cellular stress both* in vitro* and* in vivo* [5, 7–9, 14]. In contrast to ESCs and iPSCs, Muse cells exhibit telomerase activity and asymmetric growth and thus do not undergo tumorigenesis or teratoma formation when transplanted into a host organism (Figures 2(a), 2(b), 2(d), and 2(e)) [5, 7–9, 14–16]. Muse cells also exhibit a normal karyotype, as they demonstrate normal chromosome number and integrity (Figure 2(i)).

Muse cells have unique characteristics that distinguish them from other multipotent/pluripotent stem cells. They migrate to and integrate into damaged tissues to replenish cells and restore tissue function with high efficiency by single intravenous injection, as demonstrated in different animal disease models (e.g., fulminant hepatitis, muscle degeneration, skin ulcers, and brain stroke) [8–10, 16–18].

It has been proposed that Muse cells play a role in a highly conserved cellular mechanism related to cell survival and regeneration in response to cellular stress and acute injury. From an evolutionary standpoint, genes pertaining to the regenerative capacity of an organism have been lost for many species, perhaps including mammals. Muse cells could be the missing link, connecting mammals to less complex organisms, in the evolutionary chain of tissue regeneration.

The endurance of Muse cells is remarkable, evidenced by the degree of purity when cellular stress tactics are utilized for the purpose of isolation [5, 9]. This is an important feature, unique to Muse cells, which suggests their potential role in autonomous tissue regeneration. Muse cells isolated from adipose tissue, termed Muse-AT cells exhibit gene expression patterns associated with the downregulation of genes involved in cell death and survival, embryonic development, DNA replication and repair, and cell cycle [5]. Taken together, this offers insight into their evolutionary significance [5, 9]. Using a similar technology previously described [5] Gimeno et al. have recently isolated Muse-AT validating their pluripotency, growth in suspension, normal number and integrity of chromosomes, and lack of teratogenesis when injected into immunodeficient mice [14]. Furthermore, Muse-AT cells expressed high levels of TGF-*β* and possessed immunomodulatory activity as they downregulated the secretion of proinflammatory cytokines by T cells [14].

It has been speculated in primitive organisms that tumor suppressors must be transiently antagonized for autonomous regeneration to occur [19]. RNA-binding protein gene, Lin28, has been shown to maintain pluripotency and tumorigenesis in ES and iPS cells [20, 21]. In contrast, Let-7, a microRNA that regulates embryonic development, cell differentiation, and tumor suppression, opposes the action of Lin28 [21]. Lin28 and Let7 maintain a balance in molecular expression, as overexpression of Let-7 blocks Lin28 gene expression, and Lin28 expression degrades Let-7. Throughout embryonic development, we see a steady decline in levels of Lin28 expression and a simultaneous increase in Let-7 miRNAs, responsible for the suppression of cellular self-renewal of undifferentiated cells and the stimulation of cell differentiation. Of interest, ES and iPS cells exhibit a high Lin28/Let7 ratio, likely responsible for their tendency towards tumorigenesis* in vivo* (Figure 2(c)) [21]. MicroRNA Let-7 seems to be a critical upstream regulator decreasing genes involved in cell cycle division (CDCA3 and CDC16) cell differentiation (DZIP1), cellular growth and proliferation (SSR1), DNA replication factor and cancer (RFC3, RFC5, and MCM6), and cell death and survival (NUF2, BRCA1, BUB1B, and CDK6) which potentially balance cell development and oncogenesis preventing Muse-AT cells forming teratomas (Figure 2(h)). These results strongly suggest that overexpression of Let-7 in Muse cells is a putative target for further exploration. Muse cells lack strong Lin28 expression (<1000-fold than ES and iPS) (Figure 2(g)), allowing them to maintain their pluripotency [7]. In turn, elevated expression of Let-7 in Muse cells could claim responsibility for the suppression of Lin28 expression, preventing tumor formation and promoting tissue regeneration (Figure 2(f)) (unpublished data).

This molecular phenomenon regarding the balance between cell differentiation and tumor suppression is in accordance with the prominent theory regarding tissue regeneration in planarians, zebra fish, newts, and salamanders, among other more primitive species. Stem cells dictate the integral processes of postnatal regulation of growth and homeostasis in these animals, thus supporting their evolutionary significance [22]. This review details the evolution of autonomous tissue regeneration in primitive animal species and finally future avenues of investigation in mammals. We posit that pluripotent, nontumorigenic Muse cells have similar cell plasticity and capacity for tissue repair as described in animals with autonomous regeneration.

## 2. Muse Cells and Tissue Regeneration

Muse cells have a tremendous capacity to function as restoring cells for a wide range of tissues and organs. Muse cells are unique from other types of stem cells, such as somatic stem cells, neural stem cells, hematopoietic stem cells, and ESC/iPSCs due to their high capacity to home into injured/damaged tissue.

When administered intravenously, Muse cells replenished new skeletal muscle cells (human dystrophin- and paired box 7-positive cells) in a muscle degeneration mouse model [8]. Muse cells also harbor the ability to home into the liver in a fulminant hepatitis mouse model replenishing new cells and contributing to tissue repair [8]. Muse cells migrate and integrate into damaged liver in a liver fibrosis mouse model [16]. Fibrotic liver area and serum total bilirubin are decreased, while serum total albumin is increased, demonstrating functional restoration [16].

Additionally, locally injected Muse cells significantly accelerated healing of skin ulcers generated in a type 1 diabetes mouse model. The Muse-rich fraction integrated into the dermis of the mice, differentiating into vascular endothelial cells, dermal fibroblasts, and keratinocytes. Remarkably, these cells were not detected in functional regions surrounding the skin ulcers [10].

Muse cells transplanted into the rat ischemic cortex of a rat stroke model integrated into the damaged tissue and differentiated into neuronal cells. Muse cells show high graft survival and long-term engraftment in the stroke peri-infarct area as well as sensory and motor cortex with improvement in both neurological and motor functions [18].

Based on their* in vivo* capacity for tissue regeneration and functional restoration, Muse cells can be considered a promising candidate for regenerative medicine and stem cell therapy.

## 3. Autonomous Regeneration in Animals

**Figure 3:**
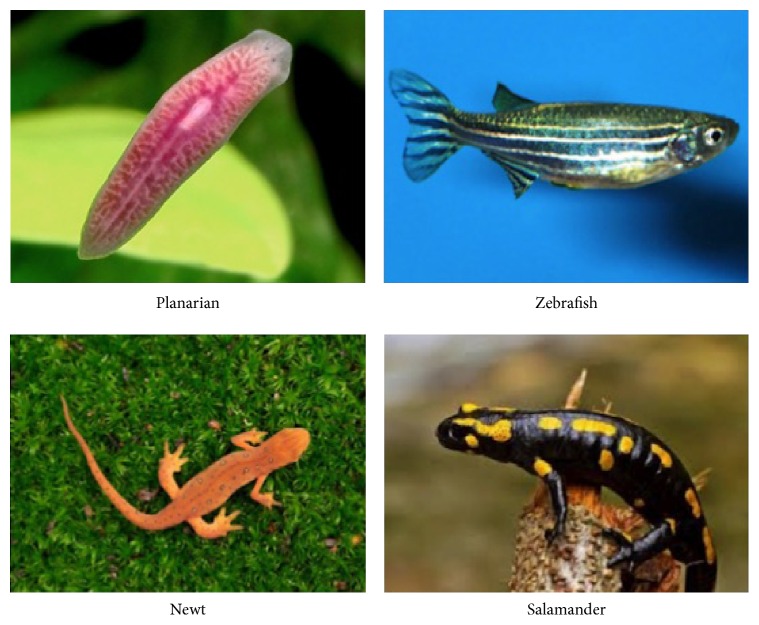
Planarians, zebrafish, newts, and salamanders have the capacity to self-regenerate damaged appendages and critical organs.

It is widely understood that species such as planarian, zebrafish, newt, and salamander have the capacity to self-regenerate damaged appendages and critical organs (Figure 3) [23]. Each species has made a significant contribution to the study of autonomous regeneration. Areas of particular interest have included limb and tail regeneration in salamanders, axolotls and Xenopus tadpoles, heart and lens in newts, gut and germ cells of Drosophila, and fin and heart of zebrafish [24]. In this mechanism, ectodermal, mesodermal, and endodermal precursors must be activated to dedifferentiate or transdifferentiate, often simultaneously, to a proliferative state in order to give rise to a fully functional limb or organ. It has been theorized that, in zebrafish, genes supporting autoregeneration are activated in response to acute injury in regenerative systems, but not in nonregenerative systems, which provides another overlap with the regenerative nature of Muse cells [5, 10]. Furthermore, cellular stress has been posited as another avenue of regenerative activation, a clear commonality with Muse cells, and their stress-induced awakening from quiescence* in vivo* [25–27]. It has been speculated that tumorigenic factors temporarily come into play at the initiation of autoregeneration, suggestive of an induction of tumor suppressor inhibitors. For example, Rb, a tumor suppressor, is inactivated in newt generation, lending insight into a vital requirement for autonomous regeneration [28].

There exists a dispute as to whether or not dedifferentiation, transdifferentiation, or a combination both is responsible for autoregeneration in nonmammalian animal species. In support of the dedifferentiation theory, it has been observed that previously quiescent cells achieve a renewed ability to divide, without retaining most of the structural markers characteristic of their previous cell type [29, 30]. Studies of Limb regeneration in* Amblystoma* larvae exhibited structural and morphological changes in cartilage cells, suggesting that dedifferentiation is the primary regenerative process [31]. Furthermore, axolotl regeneration with regard to mature muscle fibers in the tail has been shown to occur via the dedifferentiation process [32]. This is evidenced by both immunofluorescent and morphological studies [32].

Despite relatively widespread support of the dedifferentiation theory of autoregeneration, evidence has been put forth in support of the transdifferentiation theory. In salamanders, the blastema functions as the origin of limb and tail regeneration. It has been shown in contrast to previous dogma, which deemed the blastema comprised of a homogenous cell population, that the blastema is heterogeneous and contains progenitor cells that have limited plasticity due to an epigenetic memory [33]. This genetic restriction confines these cells to the production of cell types of their original lineage, preventing genuine pluripotency [33]. Additionally, pigmented epithelial cells have been shown to transdifferentiate into lens cells in newts during autoregeneration [34]. Evidence regarding the molecular intricacies of this process, beyond the scope of this chapter, suggests that cell transdifferentiation in newt lenses involves a reprogramming event during which cells are able to interconvert [35].

In summary, while dedifferentiation in some cases is irrefutable, the notion of transdifferentiation is gaining momentum as a prominent theory within the scientific community. We will now introduce the controversial role of stem cells in autoregeneration and discuss how they come into play in one of the most illuminating and thoroughly examined species in this field.

Recently, a stem cell model has been proposed as the origin of tissue regeneration in animals. In the* Xenopus* tadpole, limb regeneration occurs through preexisting precursors rather than dedifferentiation or transdifferentiation. It has been proposed that some of these precursors may indeed be adult stem cells [36, 37]. This species exhibits a process of regeneration akin to that seen in mammals, providing an additional link between mammals and less complex, yet evolutionarily significant species.

In the planarian, a species of the order Tricladida of the phylum Platyhelminthes, often characterized by their bilateral symmetry, adult stem cells are responsible for the whole-body, triploblastic regenerative capacity of these organisms [38]. Freshwater planarians have the miraculous capacity to generate an entire, functional organism from small bodily fragments [39]. Neoblasts, a term used to define adult stem cells in planarians, are a mosaic of toti-, pluri-, and multipotent, mitotically active stem cells that comprise 20–35% of the planarian cell make-up and do not form teratomas like ESCs and iPSCs, likely due to high Let7/Lin28 ratio (Figure 2(f)) [40]. Furthermore, neoblasts remain undistinguished until differentiation and their progeny have been shown to produce muscle, rhabdite cells, and germ cells [41–44].

Planarians exhibit an astonishing propensity for generation and cell plasticity. In response to the activation of the expression of signaling proteins, neoblasts migrate to the site of injury to initiate tissue regeneration and repair [45]. At the site of injury, undifferentiated cells form the regenerative blastema, and after 3-4 weeks, the amputated or injured area is restored to original morphology and function [46]. In addition, hydras show head regeneration after decapitation and show apoptotic-induced proliferation of cells [27].

Planarians have the unique capacity to shrink their body size in response to starvation via apoptotic mechanisms that have not received extensive investigation [47, 48]. This indicates a highly conserved capacity to respond to and withstand an instance of severe cellular stress that does not involve acute injury. Despite extensive investigation into the location and origin of planarian neoblasts and evidence supporting the presence of at least a percentage of pluripotent stem cells, their true potency and potential have yet to be determined and it a promising avenue for further study of autoregenerative mechanisms driven by adult stem cells.

## 4. Muse Cells and Evolution

Muse cells potentially play a critical role in both the evolutionary conservation of pluripotency and adaptations to cellular stress from planarians to humans over the course of 500 million years. Many differentially expressed genes in Muse cells isolated from adipose tissue are highly conserved, with homologues present in small organisms including yeast,* S*.* cerevisiae*,* C. elegans*,* Chlamydomonas*,* T. californica*, and* Drosophila* [5]. Thus, it is likely that Muse cells exhibit a highly conserved cellular mechanism linked to cell proliferation and survival in response to severe cellular stress, as seen in more primitive organisms and most prominently planarians [49].

Muse cells and pluripotent stem cells present in species with autonomous regeneration share many characteristics: (i) natural cells present in a quiescent state, (ii) presence in adult tissues, (iii) activation by severe cellular stress (e.g. injuries), (iv) growth in cell clusters, (v) pluripotency, (vi) cell differentiation without teratoma formation, (vii) highly efficient homing into damaged tissues, (viii) spontaneous differentiation into specific cells located in damaged tissues, and (ix) high capacity for tissue repair [5, 7, 9, 14, 15]. However, it is entirely possible that the similarities between Muse cells and pluripotent stem cells in organisms with tissue regeneration are the result of convergent rather than homologous evolution and thus are unrelated evolutionarily.

Whether Muse cells exist as naturally pluripotent cells or undergo transdifferentiation during cellular injury is unclear. Muse cells may be pluripotent in their quiescent state and may undergo cell differentiation into the desired cell type(s) under severe cellular stress during tissue repair and functional restoration processes. This negates the need to dedifferentiate as they are already pluripotent, marking the Muse cells similar to the neoblasts, the adult stem cells in planarians. In support of this hypothesis, Kuroda et al. 2010 [8] showed that SSEA-3+ Muse cells derived from bone marrow aspirate are capable of self-renewing and forming cell clusters that express pluripotent genes and are able to differentiate into three germ layers both* in vitro* and* in vivo*. The ability of these cells to display these properties without stress exposure or artificial manipulation suggests their intrinsic pluripotency, rather than as a consequence of reprogramming through cellular stress.

On the other hand, Muse cells may undergo transdifferentiation in response to severe stress conditions (e.g., cellular injury), as they become dedifferentiated and reprogrammed into pluripotent stem cells and then redifferentiate into the desired cell type(s) during tissue repair and functional restoration processes. Muse cells activated under severe cellular stress exhibit SSEA-3 in 60–90% [5, 14] of the population, and thus it is possible that Muse cells are awakened under conditions of severe cellular stress and prompted to reprogram through transdifferentiation and/or dedifferentiation. If this is the case, they undergo a form of “natural reprogramming” which negates the necessity of any exogenous genetic manipulation as seen in iPSCs and improves upon existing models [29]. This natural reprogramming may also explain the dampened expression of the Yamanaka factors present in Muse cells as compared to iPSCs [5], giving rise to their pluripotency but preventing teratoma formation. In this case, the potential transdifferentiation of Muse cells likens them to the lens cells of the newt, which also undergo transdifferentiation under stress.

Both possibilities highlight the uniqueness of Muse cells from other stem cell types, particularly iPSCs. Muse cells do not require induction of the Yamanaka factors to exhibit pluripotency but rather exhibit pluripotency in a natural state or are reprogrammed to pluripotency under severe cellular stress conditions.

Because Muse cells are likely very primitive cells involved in the evolution of cell survival in response to severe cellular stress, they offer insight into the molecular and evolutionary bases of the fascinating and tenuous phenomenon of autonomous tissue regeneration. It is anticipated that Muse cells will help to elucidate the molecular and cellular mechanisms which prevent mammals from regenerating limbs and organs, as other organisms do, revolutionizing the field of regenerative medicine.
